# An Optimized Freeze‐Dry Multimodal Workflow for Sequential Micro‐CT Imaging, Histology, and Molecular Profiling: A Use Case in Human Liver Fibrosis

**DOI:** 10.1002/advs.77406

**Published:** 2026-08-28

**Authors:** Kristijan Skok, Laura Činč Ćurić, Jernej Vajda, Silvia Schauer, Martin Wagner, Rudolf Stauber, Carolin Lackner, Karl Kashofer, Markus Plass, Giovanny Rodriguez Blanco, Ian R. Wanless, Uroš Maver, Katrin Panzitt, Boštjan Vihar

**Affiliations:** ^1^ Diagnostic and Research Institute of Pathology Medical University of Graz Graz Austria; ^2^ Institute of Biomedical Sciences, Faculty of Medicine University of Maribor Maribor Slovenia; ^3^ Division of Gastroenterology and Hepatology Department of Internal Medicine Medical University of Graz Graz Austria; ^4^ Research Unit for Translational Nuclear Receptor Research Graz Austria; ^5^ BioTechMed Graz Graz Austria; ^6^ ZLP—Zentrum für Leber‐ und Pankreaspathologie, ADK Diagnostics Vienna Austria; ^7^ Clinical Institute of Medical and Chemical Laboratory Diagnostics Medical University of Graz Graz Austria; ^8^ Department of Pathology, Queen Elizabeth II Health Sciences Centre Dalhousie University Halifax Nova Scotia Canada; ^9^ University of Maribor Faculty of Medicine Department of Pharmacology Maribor Slovenia

**Keywords:** cirrhosis, computed tomography, histology, liver fibrosis, multimodal imaging

## Abstract

Classical histology remains the gold standard for liver fibrosis assessment but is limited by destructive sectioning, 2D information, and sampling bias. Micro‐CT offers non‐destructive 3D imaging, yet conventional contrast agents can compromise tissue integrity and downstream analyses. This study establishes a resource‐efficient multimodal workflow that enables high‐quality imaging and subsequent histological and molecular testing from the same specimen. FFPE normal and cirrhotic liver tissues (*n* = 3 each) undergo an optimized micro‐CT protocol: DPBS wash, 30% eosin incubation, dehydration, snap‐freezing, and lyophilization. After imaging, tissues are rehydrated, re‐embedded in paraffin, and processed for routine histology, immunohistochemistry, RNA extraction with qPCR, and targeted NGS (800‐gene panel). The protocol yields high‐contrast, artifact‐free micro‐CT images with clear visualization of nuclei, sinusoidal networks, portal structures, and fibrotic septa (∼20x–40x optical equivalent). Volumetrics show minimal fibrosis in normal liver (2.8%) versus extensive remodeling in cirrhosis (43.5%). Morphology and antigenicity are preserved (H&E, PSR, IHC). RNA quality supports qPCR, revealing upregulation of inflammatory and cirrhosis‐related genes in cirrhosis. Targeted NGS libraries are successfully generated, with protocol‐dependent performance differences. This minimally destructive workflow maximizes information yield from limited tissue, enabling integrated imaging and molecular profiling with broad utility in pathology, biobanking, and translational research.

## Graphical abstract

Optimized freeze‐drying and eosin staining deliver artifact‐free micro‐CT images of liver tissue at near‐histological detail, then hand the same specimen back for sectioning, staining, and sequencing. Fibrotic volume distinguishes normal liver from cirrhosis, while preserved RNA reveals inflammatory gene upregulation—one biopsy, three dimensions, and a full morphomolecular readout.

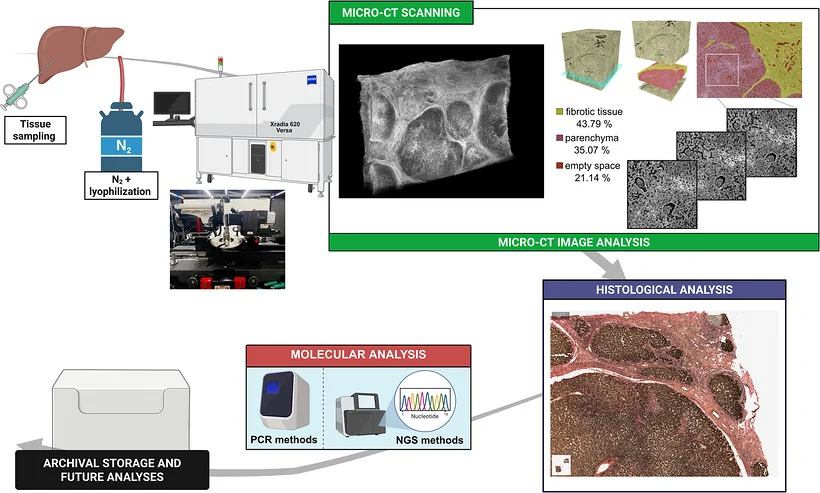

## 1 Introduction

Histological examination of tissue is a crucial diagnostic and prognostic tool used in patient management for a wide spectrum of diseases. However, classical histological tissue analysis requires sectioning, thereby destroying tissue integrity. In recent years, non‐destructive three‐dimensional (3D) imaging methods have increasingly complemented conventional histology [1, 2, 3]. Among them, micro‐CT provides high‐resolution volumetric visualization of intact tissue without sectioning artifacts [4]. Compared with standard histology, which is inherently two‐dimensional and prone to sampling bias, micro‐CT allows unlimited virtual re‐slicing in any plane and enables quantitative assessment of tissue architecture. While this approach is already established to some degree in certain disease models (e.g., pulmonary fibrosis) [1, 2, 3], its broader use in human tissues and translational pathology to evaluate fibrosis remains limited. Nevertheless, the ability to visualize fibrotic networks and vascular remodeling in situ [3, 5, 6] highlights micro‐CT as a powerful and truly complementary tool for morphological tissue analysis [7]. This is especially the case for liver diseases. Liver diseases are frequently characterized by a heterogeneous spatial distribution of pathological alterations, rendering conventional two‐dimensional histological assessment susceptible to sampling bias [8, 9]. Furthermore, once tissue is sectioned and stained, it is no longer available for additional analyses, limiting multimodal diagnostic and translational research approaches, particularly when only limited human tissue is available.

Different contrast‐enhancement strategies have been developed to overcome the inherently low X‐ray attenuation of soft tissues. Commonly used approaches include diffusible iodine‐based contrast agents (e.g., Lugol's iodine), phosphotungstic acid (PTA), phosphomolybdic acid (PMA), osmium tetroxide, eosin‐assisted staining, and propagation‐based phase‐contrast X‐ray imaging (XPCT). These methods differ substantially in staining kinetics, tissue penetration, achievable image contrast, reversibility, and compatibility with downstream histological and molecular analyses [5, 10, 11]. In addition to absorption‐based contrast enhancement, propagation‐based and other phase‐contrast micro‐CT techniques have emerged as powerful alternatives for imaging soft tissues, enabling excellent visualization of weakly absorbing biological structures without the need for exogenous contrast agents [12, 13]. These approaches have demonstrated impressive performance for virtual histology and 3D tissue characterization. Most published studies focus on optimizing image acquisition and analysis rather than on establishing tissue preparation workflows compatible with comprehensive downstream histopathological and molecular investigations. However, recent developments have increasingly focused on integrating 3D imaging with histology and digital pathology to create multimodal tissue analysis pipelines capable of combining structural, morphological, and molecular information from the same specimen [14].

Consequently, no single protocol is universally optimal, and the choice of preparation depends on the intended application. Nevertheless, iodine‐ and heavy‐metal‐based staining protocols have become the most widely used approaches for laboratory soft‐tissue micro‐CT despite concerns regarding tissue preservation and compatibility with downstream analyses [15]. In parallel, XPCT [16] has demonstrated the potential of virtual histology by providing near‐histological 3D visualization of intact tissues without exogenous contrast agents [17, 18, 19, 20]. These studies have highlighted the considerable value of volumetric pathology and correlative imaging, but rely on highly specialized infrastructure that remains inaccessible to most pathology laboratories. There remains a need for practical, laboratory‐based workflows that can be readily integrated into routine pathology while remaining compatible with downstream analyses [21, 22]. With the emergence of advanced molecular technologies, next‐generation sequencing, spatial transcriptomics, and spatial proteomics, preservation of tissue for multiple sequential analyses has become increasingly important for both diagnostic pathology and translational research [23, 24]. Integrating micro‐CT with histological and molecular analyses enables comprehensive characterization of valuable tissue specimens, particularly diagnostic biopsies and rare clinical samples where tissue availability is limited, and every downstream analysis is of high importance [10, 25].

Therefore, this study aimed to establish and validate a resource‐efficient multimodal workflow that enables sequential micro‐CT imaging, routine histology, immunohistochemistry, and molecular analyses using the same tissue specimen. Human liver tissue was selected as a representative use case because of its heterogeneous architecture, the spatial variability of fibrosis, and the need for comprehensive tissue characterization. Although individual components of the present workflow, including laboratory micro‐CT, snap‐freezing, lyophilization, histological processing, and molecular analyses, have been reported previously, they have generally been developed and applied independently. To the best of our knowledge, no previous study has established and validated a sequential tissue‐preparation workflow that combines these methodological steps while preserving compatibility with high‐resolution micro‐CT imaging, histopathology, immunohistochemistry, RNA‐based analyses, and targeted next‐generation sequencing on the same tissue specimen.

## 2 Materials and Methods

### 2.1 Tissue Processing Overview

**FIGURE 1:**
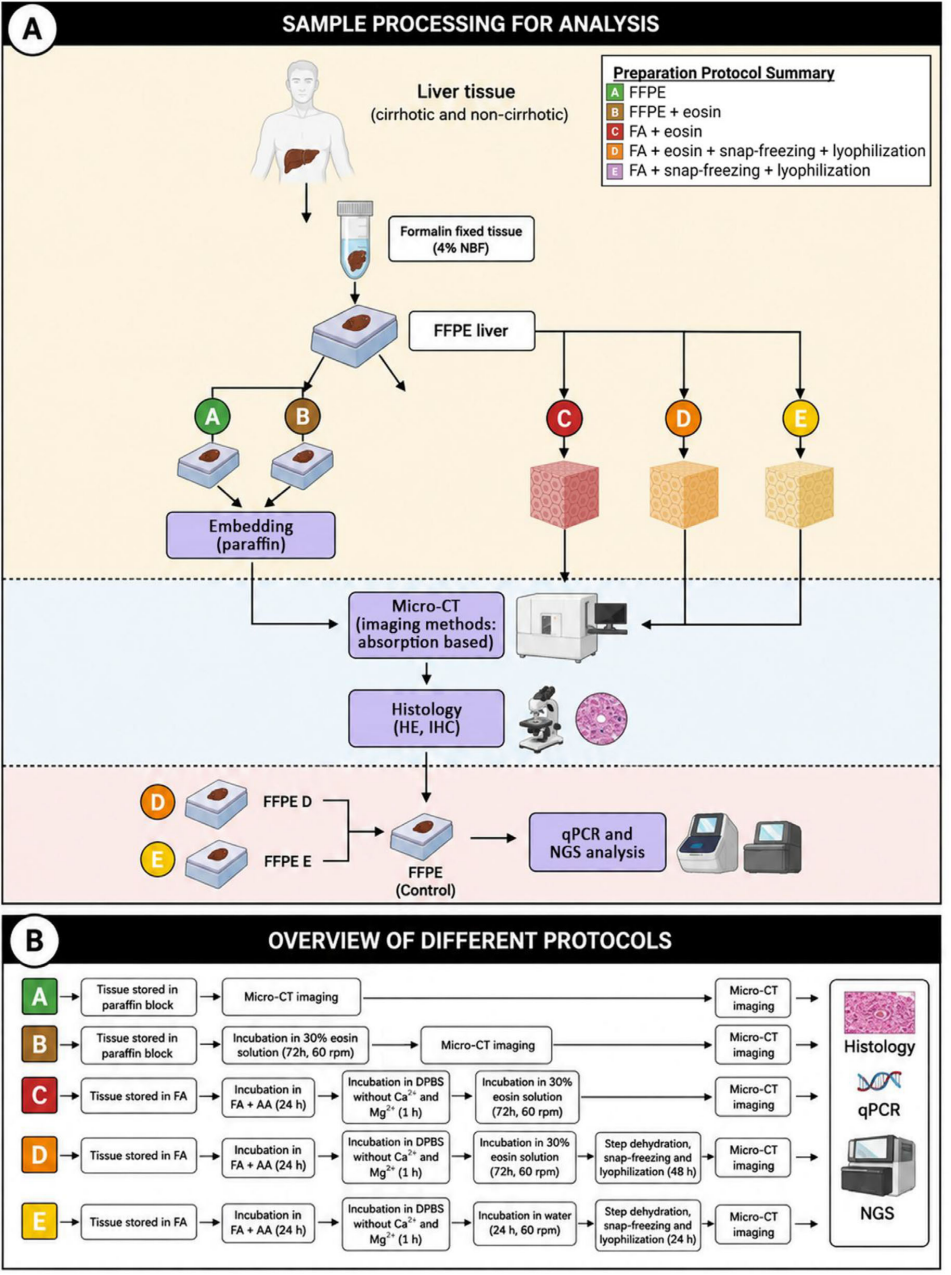
Graphical workflow summary (A) and description of the utilized protocols (B). (A) Following formaldehyde (FA) fixation, samples were processed using different workflows. Protocol A: FA‐fixed sample embedded in paraffin. Protocol B: Protocol A followed by eosin staining. Protocol C: FA‐fixed sample acidified with glacial acetic acid (AA), washed in PBS, and stained with eosin. Protocol D: Protocol C followed by ethanol dehydration, snap freezing, and lyophilization. Protocol E: FA‐fixed sample acidified with AA, washed in PBS and ultrapure water, followed by dehydration, snap freezing, and lyophilization (yellow area). Blue area: Micro‐CT imaging. Light green area: Light microscopy was used to assess tissue morphology and compatibility with conventional histopathological analyses. Pink area: Next‐generation sequencing (NGS) on cirrhotic and normal liver specimens from protocols D and E. (B) Simplified overview of the individual processing protocols. AA, glacial acetic acid; PBS, phosphate‐buffered saline; rpm, revolutions per minute. Created using Biorender.

A general overview of tissue processing and the following methodology is shown in Figure 1, with Figure 1A showing the tissue processing step and Figure 1B providing an overview of the different handling protocols.

### 2.2 Human Tissue Samples

Liver tissue was obtained from anonymized autopsy cases at the Medical University of Graz, including both histologically normal and cirrhotic samples. Cases were screened for fibrosis based on autopsy reports. Only specimens without advanced autolysis were included. Tissue samples were obtained from six patients, three patients with a cirrhotic liver, and three with a normal (non‐cirrhotic) liver. All procedures were conducted in accordance with the Declaration of Helsinki and approved by the local ethics committee (EK 1174/2024). To stabilize the specimens, these were fixated by incubation in 4% Formaldehyde (1.00496, Sigma Aldrich, Darmstadt, Germany) and stored at room temperature, protected from light. Before further processing, tissue samples were hand‐cut using a scalpel to prepare specimens approximately 3 × 3 × 3 mm in size.

### 2.3 Tissue Preparation Protocols

#### 2.3.1 Eosin Staining

To test improved contrast, the samples were subjected to eosin staining as described by Busse et al. [3]. Briefly, the samples were acidified by incubation in a solution prepared using 9.5 mL of 4% Formaldehyde solution containing 0.5 mL glacial acetic acid (AA) 1.00063, Sigma Aldrich, Darmstadt, Germany) for 24 h at room temperature. The specimens were washed by a 1‐hour incubation in DPBS (D8537, Sigma Aldrich, Darmstadt, Germany) at room temperature, followed by soaking in a 30% (wt.) Eosin Y (E4009, Sigma Aldrich, Darmstadt, Germany) solution in ultra‐pure water for 24 h at room temperature while agitated on an orbital shaker at 60 RPM. Finally, the samples were patted dry with lint‐free tissue and stored in Eppendorf tubes under ethanol vapor.

#### 2.3.2 Dehydration, Lyophilization, and Paraffin Embedding

To dry the specimens were subjected to a graded ethanol dehydration series (50%, 60%, 70%, 80%, 90%, 96%, and 100%), with each step lasting 30 min. Dehydrated samples were snap‐frozen in liquid nitrogen, then lyophilized for 24 h and stored in dry microcentrifuge tubes. The scanned tissues were re‐embedded in paraffin blocks. Thereafter, they were cut into tissue sections (3 µm) and stained with a variety of stains (see Section 2.4) [26].

### 2.4 Micro‐CT

#### 2.4.1 Scanning Procedure

Measurements were performed with a Zeiss Xradia 620 Versa 3D X‐ray microscope. Before scanning, each sample was carefully placed in a 1.5 mL microcentrifuge tube, between two pieces of Styrofoam to keep it in place. The centrifuge tube was closed and attached to the sample mount using double‐sided adhesive tape (Figure S1). Imaging was performed using 4× and 20× objectives at 50 kV and 4.5 W, without an additional filter. For the 4× objective, the exposure time was set to 1 s with binning of 2, acquiring 1601 projections at 1 image per projection. The source‐to‐sample and sample‐to‐detector distances during acquisition were set to 17 mm, which, at 4× magnification, resulted in a cone angle of 5.26° and a pixel size of 3.31 µm. For the 20× objective, the exposure time was 5 s with binning of 2, and 2401 projections were acquired, with 1 image per projection. The source‐to‐sample and sample‐to‐detector distances during acquisition were set to 17 mm, which, at 20× magnification, yielded a pixel size of 0.68 µm. After the imaging procedure, the scans were reconstructed and merged using Zeiss' proprietary reconstruction software, followed by additional image processing, segmentation, 3D visualization, and analysis using Dragonfly software (Object Research Systems, Montreal, Canada). Manual annotation of fibrotic regions was independently reviewed by three experienced liver pathologists (C.L., S.K., and I.W.) to ensure accurate identification of fibrotic tissue before neural network training and validation.

#### 2.4.2 Specimen Processing Strategy Optimization

To identify an optimal preparation strategy for micro‐CT imaging of liver tissue while preserving integrity for downstream analyses, five protocols were evaluated (Figure 1B):

- Protocol A–Paraffin embedding: The fixated samples underwent paraffin embedding followed by direct scanning.
- Protocol B–Paraffin embedding + eosin staining: Samples were prepared as per Protocol A with the addition of staining by incubating in 30% wt. Eosin solution for 72 h at room temperature with gentle agitation before scanning.
- Protocol C–Fixed wet tissue + eosin: Formaldehyde/acetic acid–fixed specimens were washed in DPBS (1 h, RT), incubated in 30% eosin (72 h, RT), and scanned in hydrated state.
- Protocol D–Fixed wet tissue + eosin + dehydration/lyophilization, followed by dehydration and lyophilization.
- Protocol E–Fixed wet tissue dehydration and lyophilization (no eosin): Similar to Protocol D, but without eosin incubation. Fixed liver specimens were dehydrated, snap‐frozen, and lyophilized, producing high‐contrast, artifact‐free images.

#### 2.4.3 Micro‐CT Image Segmentation

Image processing of the scans was performed using Dragonfly Pro Software (Version 2022.1, Build 1259) with supervised artificial intelligence (AI)‐ supported segmentation, based on initial manual annotation of the images, to separate functional parenchyma, fibrotic tissue, and empty spaces in the specimens. The computer on which the analysis was performed uses a Ryzen 9 9950 × 3D CPU (AMD, Santa Clara, CA, USA), a GeForce RTX 5090 GPU (NVIDIA, Santa Clara, CA, USA) with 32 GB VRAM and 128 GB of DDR5 RAM. The segmentation wizard was employed to train a 3D U‐Net deep learning model from scratch based on 25 manually segmented frames. The number of manually segmented frames is consistent with established practice for U‐Net‐based segmentation in tomographic imaging. The original U‐Net achieved state‐of‐the‐art performance with only 20 to 35 annotated images, having been specifically designed to learn from small datasets through aggressive data augmentation [27]. Its volumetric extension, the 3D U‐Net, was explicitly developed for sparse annotation and has been shown to generalize from only a few annotated slices to dense 3D segmentations, as biomedical volumes contain repetitive structures with intrinsic variation [28]. This principle has been validated in synchrotron micro‐CT, where a U‐Net trained on sparse scribble‐level annotations across only four volumes achieved high Dice scores [29] and quantitative analyses on CT segmentation confirm an exponential‐plateau relationship between training set size and Dice score, with performance saturating well before what is conventionally considered a large dataset [30]. The model was trained on 3D data with a cubic patch size of 32, depth level of 4, and 100 epochs, with data augmentation set to 10. Three classes were defined: class 1–representing parenchyma, class 2–fibrotic tissue, and class 3–empty space (e.g., lumen of vasculature). This AI‐based approach was chosen to ensure accurate and consistent classification of tissue structures while significantly reducing manual segmentation time [31, 32]. The only processing performed for image enhancement in this work consists of rotation, cropping, and global contrast adjustment, all of which are part of the regular image handling in the mentioned software.

#### 2.4.4 Processing‐Related Sample Deformation

To quantify tissue shrinkage across protocols that include dehydration and lyophilization, additional CT scans of one sample each were performed for Protocols D and E. CT scans were acquired at three time points: before dehydration, after dehydration, and after lyophilization, using identical imaging settings. Scanning was performed at a source‐to‐sample distance of 40 mm and a sample‐to‐detector distance of 125 mm, with a source voltage of 60 kV, 6.5 W of power without the filter, camera binning of 2, and 1s of exposure time per frame. Segmentation was performed in Dragonfly 3D World 2025.1 (Comet Technologies, Montreal, QC). An initial region of interest (ROI) was defined by intensity thresholding using the Define Range function, and small disconnected components were removed using slice‐wise island filtering (Process Islands in 2D, 8‐connected, threshold: 5000 voxels). Internal voids were filled across all three axes and volumetrically (Fill Inner Areas in 2Dx/y/z and 3D). The ROI boundary was then regularized by sequential morphological closing, dilation, and erosion (equal iteration counts), and any residual artifacts were corrected manually using the ROI Painter. The unmodified initial ROI was retained as a reference for verification at each step. Tissue volume was derived from the final refined ROI voxel count. The described sequence ensured that the bounding volume was measured, including internal pores, which could not be differentiated in the hydrated sample.

### 2.5 Histological Analysis and Staining

The sections were stained using standard histological staining procedures (e.g., Hematoxylin and Eosin). For testing antigenicity, immunohistochemistry for HepPar‐1 (Dako, cat# M7158) and Collagen IV (Biozol Diagnostica, Clone Isotype IgG1; used RTU) was applied.

Briefly, 3 µm‐thick sections were deparaffinized in xylene and rehydrated with graded ethanol. For hepatocyte detection, the sections were subjected to antigen retrieval in a microwave in BOND Epitope Retrieval Solution 2 (Leica, AR9640) and subsequently incubated for 60 min with a monoclonal mouse antibody to human hepatocytes (DAKO M7158) at a 1:200 dilution. The reaction was visualized using Dako REAL EnVision Detection System (DAKO K500711‐2) according to the manufacturer's instructions, and all sections were counterstained with hematoxylin. For the negative control, the primary antibody was omitted.

### 2.6 qPCR Analysis

A total of 6 liver patient samples, three cirrhotic and three healthy liver tissue specimens, were embedded in paraffin, whereas tissue from the same patients was freeze‐dried as per protocol D and E and analyzed (see Section 2.3.2). All patient samples were embedded in paraffin after the CT analysis. 8–10 slices per paraffin block were cut with a microtome (HistoCore rotational microtome, Leica), depending on the size of the tissue (app. 0.7 cm × 0.7 cm), with a thickness of 10 µm, and were cut from each paraffin block and used for RNA isolation. RNA isolation was performed with innuPREP FFPE total RNA kit (Innuscreen, #845‐KS‐2050010) as per the protocol. Elution was done in 30 µl of deionized water. Samples were dried in a vacuum centrifuge and resuspended in 10.5 µL of deionized water. RNA concentration was measured on a NanoDrop spectrophotometer (Thermo Fisher Scientific, Waltham, MA, USA). RNA integrity was assessed on a TapeStation (Agilent), with the corresponding electropherograms shown in Figure S3. A 300 ng of RNA were reverse transcribed (LunaScript RT SuperMix Kit; NEB #E3010) as per the manufacturer's instructions. qPCR was performed on a CFX Opus 384 (Biorad). cDNA was diluted 1:5, and NEB Luna universal qPCR Master Mix (NEB, #M3003S) was used for qPCR. 5 primers were used for qPCR: APOA1 (fw: 5’ ACTGTGTACGTGGATGTGCTCAA 3’; rev: 5’ AGTTGTCAAGGAGCTTTAGGTTTAGC 3’); MCP1 (fw: 5´ AGAATCACAGCAGCAAGTGTCC 3´; rev: 5´ TCCTGAACCCACTTCTGCTTGG 3´); CRP (fw: 5’ AACGAAGCCTCTCAAAGCCTT 3’; rev: 5’ CTCTTGGTGGCATACGAGAAAAT 3’); MMP9 (fw: 5’ TGTACCGCTATGGTTACACTCG 3’; rev: 5’ GGCAGGGACAGTTGCTTCT 3’) and 36b4 (fw: 5’GTCACTGTGCCAGCTCAGAA3’; rev: 5’ TCAATGGTGCCTCTGGAGAT 3’). mRNA levels were normalized to 36b4. Analysis was performed with the 2^− ΔΔCT^ method.

### 2.7 NGS Analysis

Six FFPE tissue samples were selected for analysis. These consisted of one cirrhotic tissue sample on which the different imaging protocols were compared (untreated; protocol D and protocol E) as well as 1 normal tissue sample (untreated; protocol D and protocol E). Genomic DNA was extracted from all specimens with a Maxwell RSC FFPE Plus DNA kit (Promega #AS1720) [33]. DNA concentration was measured fluorometrically with the QuantiFluor ONE dsDNA kit (Promega #E4871) on a Quantus Fluorometer (Promega). DNA integrity was assessed on a TapeStation (Agilent), with the corresponding electropherograms shown in Figure S2. Library preparation was performed with 50 ng of DNA as starting material, followed by hybrid‐capture enrichment according to the SureSelect XT HS2 DNA Reagent kit protocol (Agilent). Sequencing was performed using a custom 800‐gene pan‐cancer panel (Agilent). Libraries were sequenced on a Nova‐seq instrument (Illumina). Variant calling was performed using three independent software options for variant calling. These were Mutect, VarDict, and Strelka software, and allelic frequencies of germline variants were compared between the three cirrhotic and normal tissue samples, respectively.

### 2.8 Statistical Analysis

Statistical analysis for the QPCR results was performed with Prism 10. Significance was evaluated with One‐Way ANOVA and was corrected for multiple comparisons using the Tukey Test. A *p*‐value of <0.05 was considered statistically significant.

## 3 Results

### 3.1 Optimization Of Protocols is of Paramount Importance for the Success of Scanning

**FIGURE 2:**
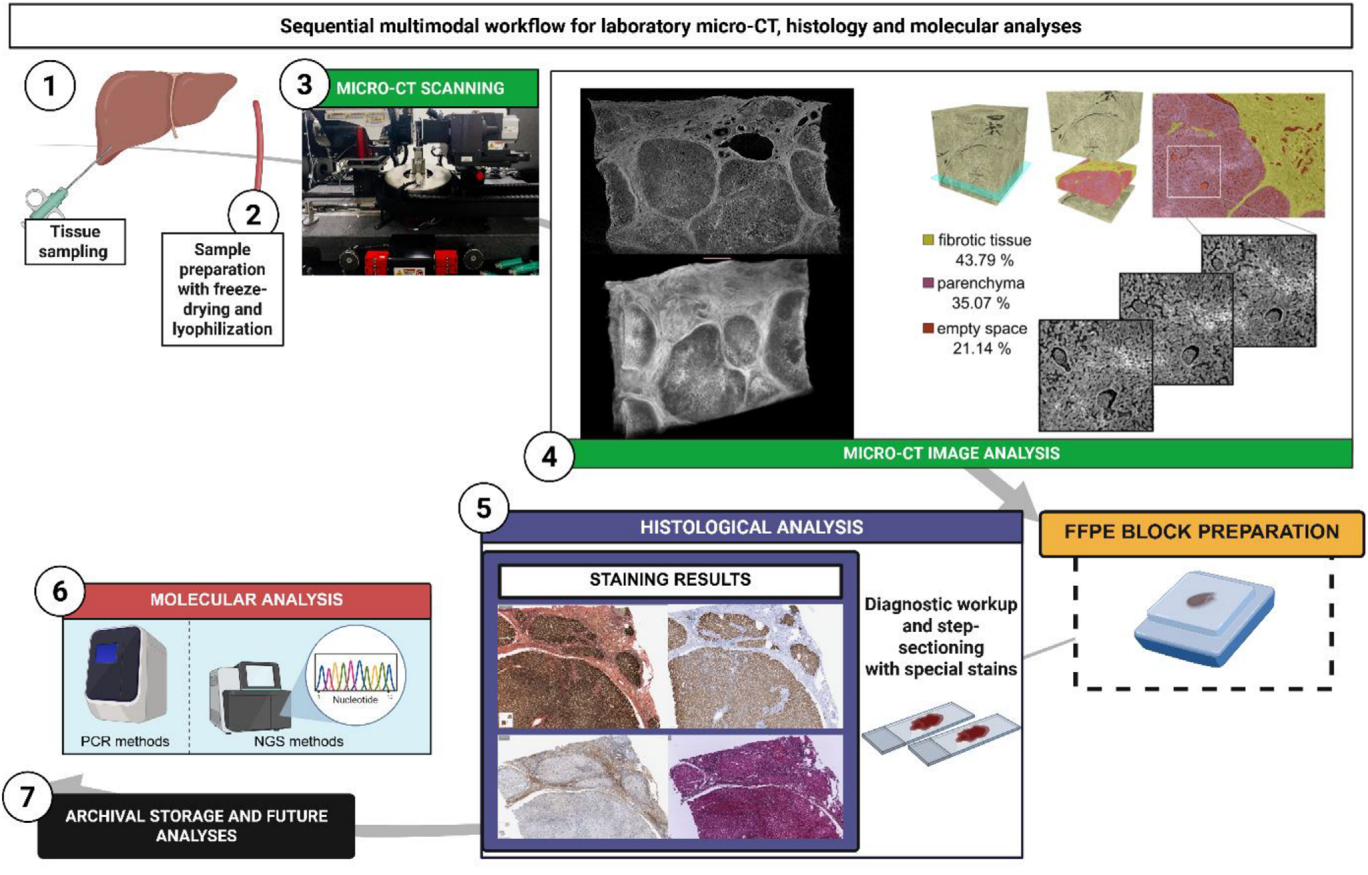
Sequential multimodal workflow for micro‐CT, histology and molecular analyses. (1) Liver tissue is obtained by biopsy or surgical resection. (2) Samples are prepared by a special protocol (including freeze‐drying and lyophilization) prior to imaging. (3) The intact specimen undergoes micro‐CT scanning to obtain high‐resolution 3D datasets. (4) Micro‐CT images are reconstructed and analyzed. After imaging, a conventional FFPE block is prepared from each specimen. (5) The same tissue specimen is subsequently used for routine histopathological evaluation, including hematoxylin and eosin staining, special stains, immunohistochemistry, and diagnostic step sectioning as required. (6) The remaining tissue is available for downstream molecular analyses, including quantitative PCR (qPCR) and targeted next‐generation sequencing (NGS). (7) Finally, the FFPE tissue block is archived. Created using BioRender.

A schematic of the integration of micro‐CT into a clinical workflow in tissue‐scarce settings is shown in Figure 2.

**FIGURE 3:**
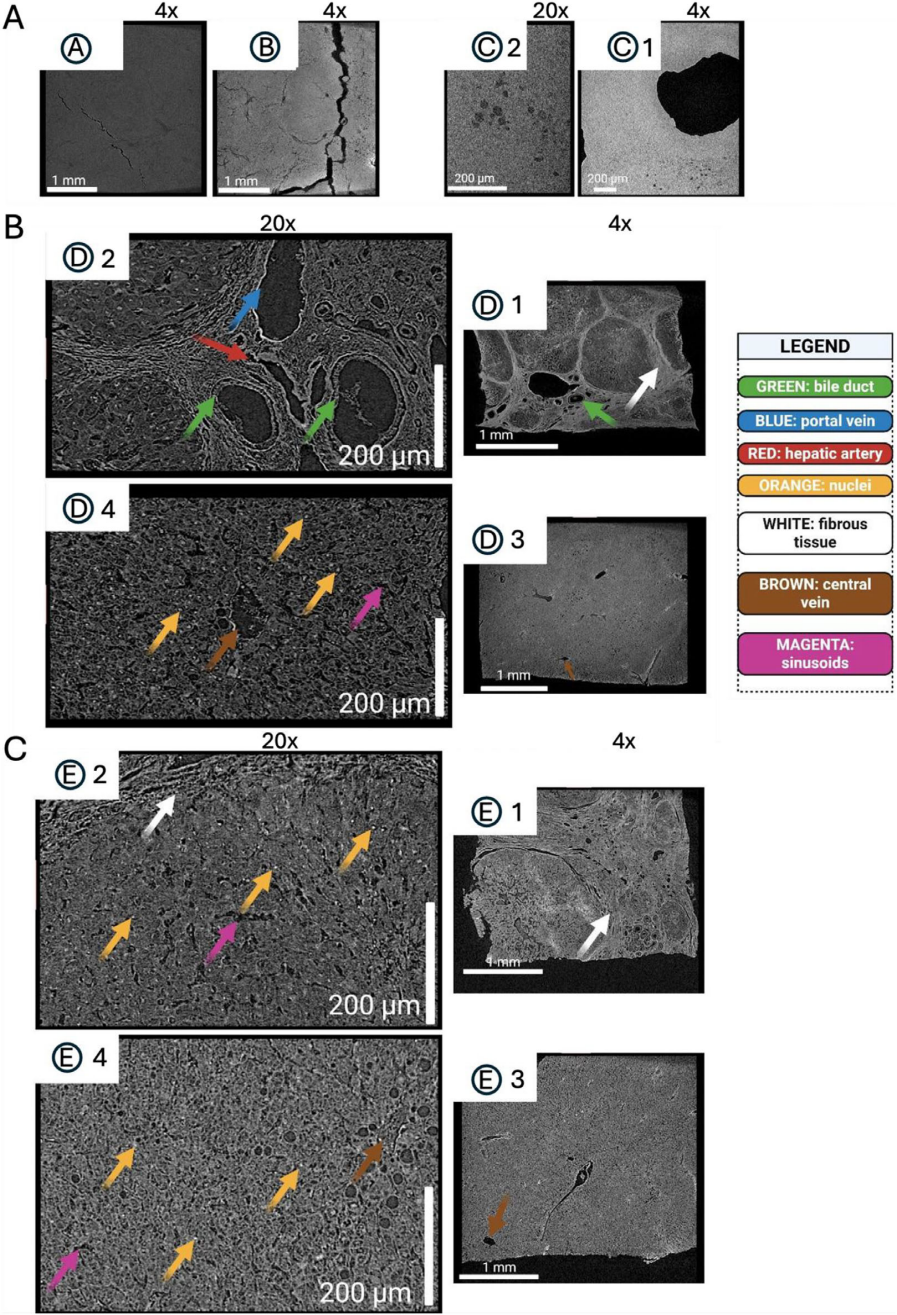
X‐ray micrographs obtained from CT reconstructions after scanning of samples prepared with all protocols. (A) Protocols A, B, and C (4×, 20×) show a somewhat comprehensible liver architecture but almost no visible details. (B) D_1 (4×) and D_2 (20×) show cirrhotic liver tissue. D_3 (4×) and D_4 (20×) illustrate preserved liver architecture. Green arrows indicate bile ducts; white arrows indicate the surrounding fibrous tissue. Orange arrows point to hepatocytes with visible nuclei, and central veins and sinusoids are marked by brown and magenta arrows, respectively. D_3 provides a low‐magnification overview of the tissue block. (C) The E series (4×, 20x) highlights detailed parenchymal organization. E_1 represents an overview(4×) of the cirrhotic liver and E_3 of the healthy liver. The white arrow indicates fibrous tissue, and the brown arrow indicates the central vein. E_2 and E_4 provide the corresponding 20x magnifications. In E_2, the white arrow indicates fibrous tissue, hepatocytes with nuclei are indicated by the orange arrow, and sinusoids by the magenta arrow. E_4 demonstrates central veins (brown arrow), hepatocytes (orange arrows), and sinusoids (magenta arrow). Protocols: A: FFPE; B: FFPE+eosin; C: formalin+eosin; D: formalin+snap‐freezing+eosin+lyophilization; E: formalin+snap‐freezing+lyophilization. Scale bars: 1 mm (low magnification), 200 µm (high magnification). Created using Biorender.

To find the optimal tissue preparation strategy for micro‐CT imaging of liver tissue followed by downstream immunohistochemical and molecular analyses, we compared five protocols, summarized in Table 1. The resulting images are presented in Figure 3 and Video S1.

**TABLE 1:** Liver tissue preparation protocols for micro‐CT imaging.

Protocol	Paraffin block (PT)	Eosin staining	Use of AA and FA	Dehyd. & Lyophil.	Processing burden	Outcome
A	✓	—	—	—	None	Poor cellular resolution.
B	✓	✓	—	—	Low	Improved cytoplasmic contrast; however, features are difficult to identify.
C	—	✓	✓	—	Moderate	Limited resolution, tissue distortion.
D	—	✓	✓	✓	High	Improved contrast and integrity vs. A, B, C, histological features (sinusoids, portal triad, central vein, fibrotic septa) are clearly visible. Allows downstream processing.
E	—	—	✓	✓	Moderate	Like D, this approach features a simpler preparation protocol that also facilitates downstream processing.

*Abbreviation*: dehyd., dehydration; lyophil., lyophilization; PT, primary tissue; AA, glacial acetic acid; FA, formaldehyde.

The protocols for testing paraffin‐embedded tissue specimens (A and B), which are based on studies by Eckermann et al. [17] and Jonigk et al. [34], have not yielded comparable results. It should be noted that both studies employed synchrotron‐based phase‐contrast X‐ray tomography, whereas the present study utilized a Zeiss Xradia Versa 620 nano‐CT system with absorption‐based imaging and propagation‐based phase‐contrast enhancement. These differences in imaging modality and contrast mechanism likely account, at least in part, for the discrepant performance of paraffin‐embedded specimens. Under the preparation and acquisition conditions used in the present study, FFPE specimens exhibited lower image contrast and preparation‐related structural artifacts compared with those from the lyophilization‐based protocols. Optimized FFPE preparation, mounting, and acquisition strategies have been shown to substantially improve image quality and reduce such artifacts in laboratory micro‐CT studies. While no physical damage in the form of cracks was observed, protocol C, which resulted in an eosin‐stained, wet specimen, showed negligible contrast and resolution of cellular structures. Specimen prepared using protocol D, which has the added dehydration and lyophilization steps (protocols C and D were both adapted from Busse et al. [3], who described a similar method for murine kidneys), showed good contrast and well‐defined cellular structures in the micro‐CT scans, revealing that protocol D is a suitable method for obtaining volumetric histological information (of at least comparable quality and to a certain extent diagnostic potential for certain questions) about liver specimens. However, the potent staining procedure limits subsequent downstream analysis.

**FIGURE 4:**
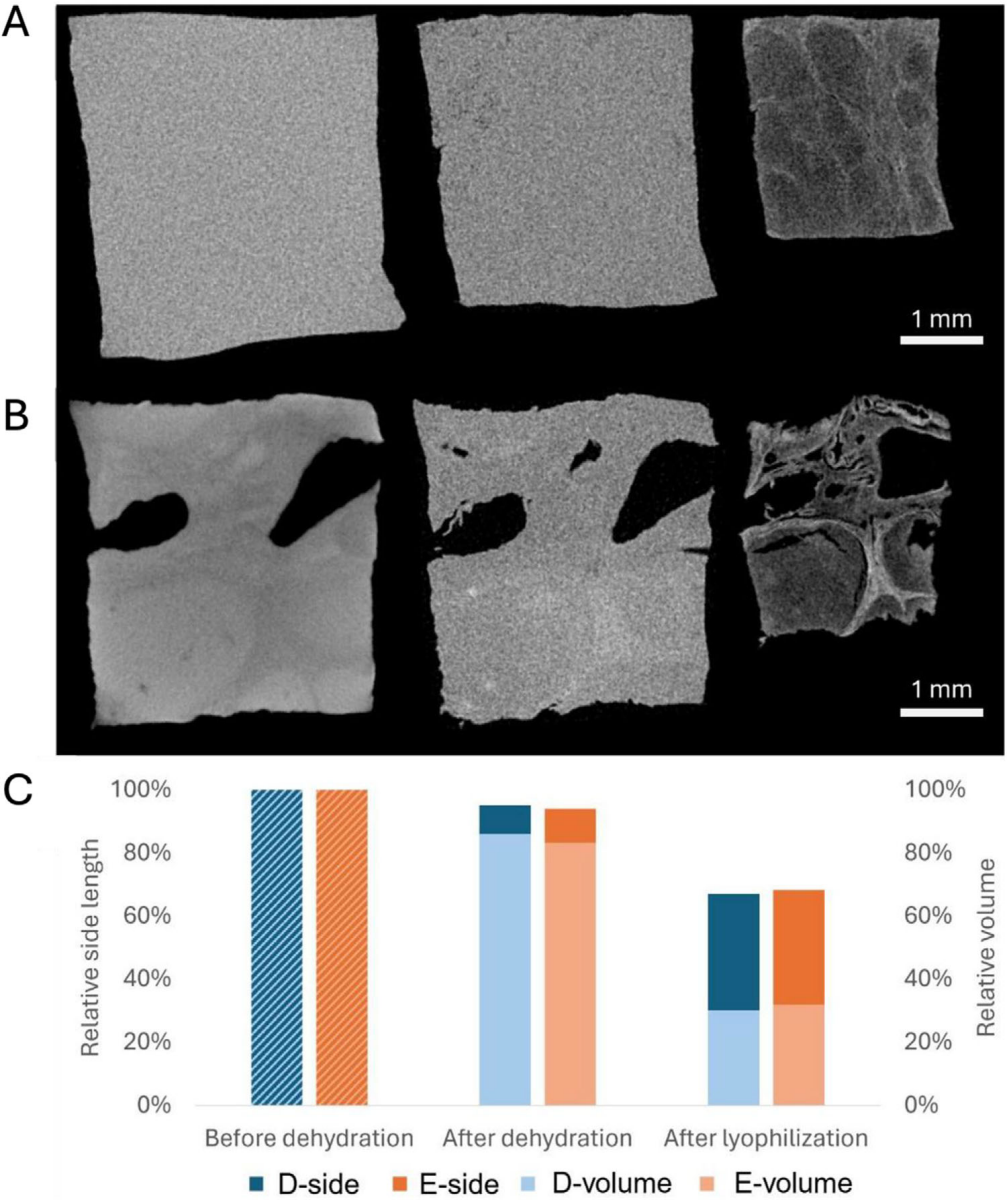
Process‐related shrinkage of the liver samples. (A) shows CT scans of the same sample undergoing protocol E before dehydration (left), after dehydration (middle), and after lyophilization (right). (B) shows the same scanning time points for a sample undergoing protocol D. (C) shows the calculated sample shrinkage at different time points. The chart shows the changes in total volume (light color) and average side length (dark color) for the respective samples undergoing protocols D (blue) and E (orange). *Source*: own.

Through sequential CT scanning and segmentation of the same sample before and after dehydration, as well as after lyophilization, changes in sample size due to processing were determined for protocols D and E. Segmentation of the CT scans allowed quantitative volumetric assessment of the bounding cuboid, which shows progressive shrinkage across both protocols, as shown in Figure 4. After dehydration, the samples retained 86% (protocol D) or 83% (protocol E) of their volumes. Lyophilization resulted in a more dramatic volume change, with the samples retaining 30% (D) or 32% (E) of their volume. Assuming isotropic shrinkage, the calculated average side length (the cube root of the measured volume) would translate to 95% (D) or 94% (E) length retention after dehydration and 67% (D) / 68% (E) after lyophilization. While processing‐related shrinkage was considerable (approximately one‐third of the original side length), the overall shape was maintained for the sample following protocol E (Figure 4A); slight deformations of the shape were visible in the sample following protocol D, which are likely related to the large proportion of vasculature (empty spaces) contained in this particular sample (a hilar tissue specimen). The sample undergoing protocol D also shows improved contrast across all processing stages, enabling approximation of the internal structure. From the distribution of hollow spaces and fibrotic tissue across all three processing stages, we conclude that while the sample size is reduced and slight deformation is observed, the internal structure and component proportions (parenchyma, fibrotic tissue, empty space) are maintained throughout the process.

As the greatest impact on the obtained image quality was correlated with tissue dehydration and lyophilization, a final protocol (E) was tested to evaluate whether this processing step was sufficient for histologically informative CT imaging. As shown in Figure 3, tissue samples prepared using protocol E reveal the histological features (central vein, sinusoids, portal triad of hepatic lobules) and partially cellular features (nuclei) relevant to the pathological analysis of liver tissue. As this protocol provided the best image quality (comparable to protocol D) and was implemented without staining or embedding, it was selected for further CT image analysis.

### 3.2 AI‐Supported Segmentation Allows for Accurate Quantification of Fibrosis in 3D Micro‐CT Models

**FIGURE 5:**
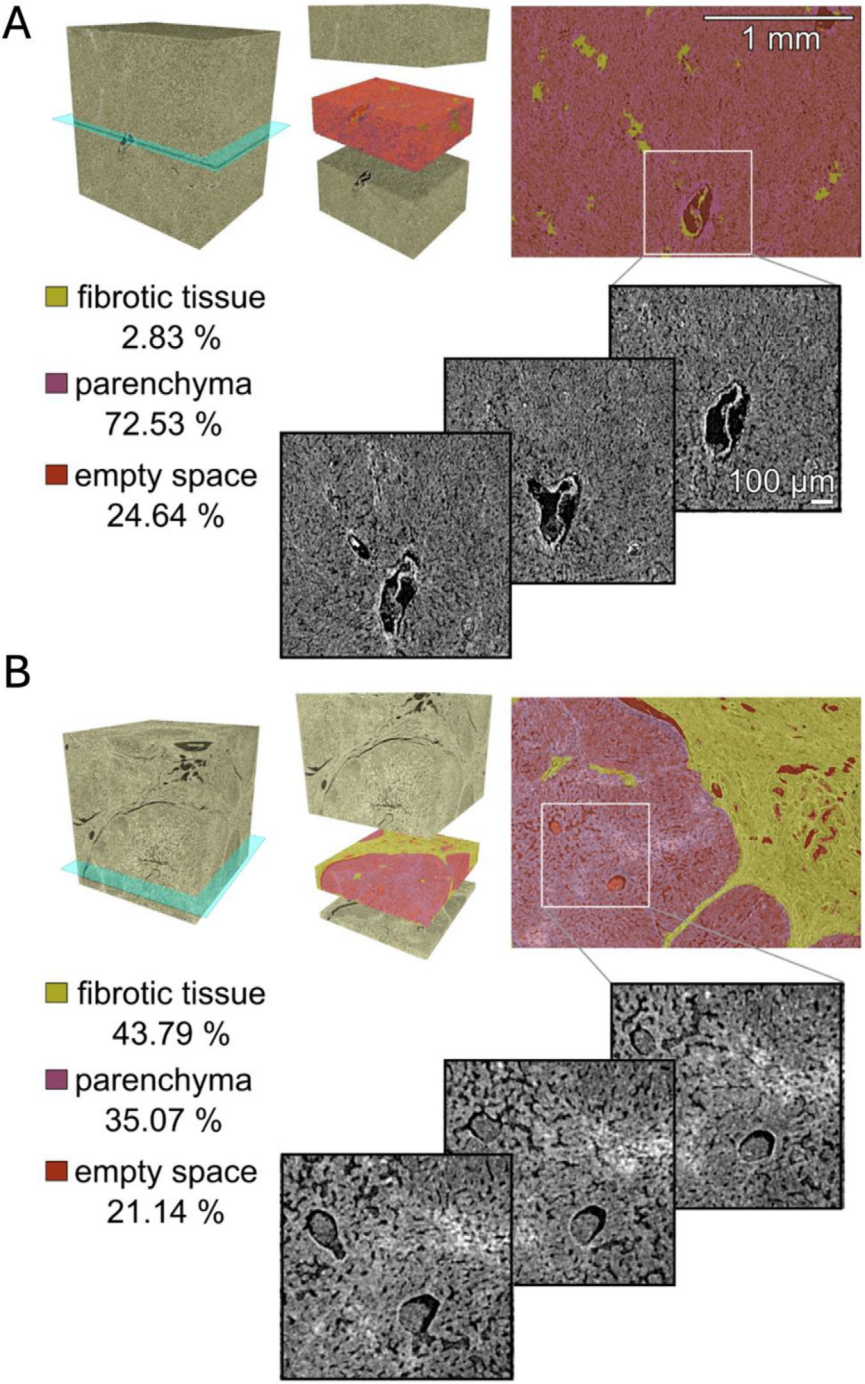
Automated fibrosis quantification in cirrhotic and healthy liver. AI‐assisted segmentation showing classification into parenchyma (nodules), fibrotic tissue, and empty space (vascular lumina) within healthy (A) and cirrhotic (B) liver tissues. *Source*: own.

After identifying the most suitable strategy for sample preparation, healthy and cirrhotic liver samples were prepared using protocol E and scanned at 4× and 20× magnification. Sample images of the scans are shown in Figure 3. Manual annotation of fibrotic regions was independently reviewed by three experienced liver pathologists (C.L., S.K., and I.W.) to ensure accurate identification of fibrotic tissue before neural network training and validation. AI‐assisted segmentation was applied to quantify structural features in the reconstructed volumes as described in section 2.3.3. The results are shown in Figure 5. The deep learning model successfully classified parenchyma, fibrotic tissue, and empty spaces (vascular lumina). As a proof of concept, volumetric analysis showed marked fibrosis in cirrhotic liver tissue (43.79%) compared with minimal fibrosis in normal liver (2.83%), which is qualitatively consistent with the corresponding histological assessment (Figure 5). These results demonstrate the feasibility of objective, 3D fibrosis quantification within intact liver specimens. Furthermore, one can appreciate that during cirrhotic remodeling, the borders between fibrous septa and parenchyma are not sharply delineated (for example, pushing border phenomena) but are rough, with jagged contours.

Video S1 shows the whole process of the micro‐CT scanning and three‐dimensional segmentation of human liver tissue.

### 3.3 Histological Tissue Comparison

**FIGURE 6:**
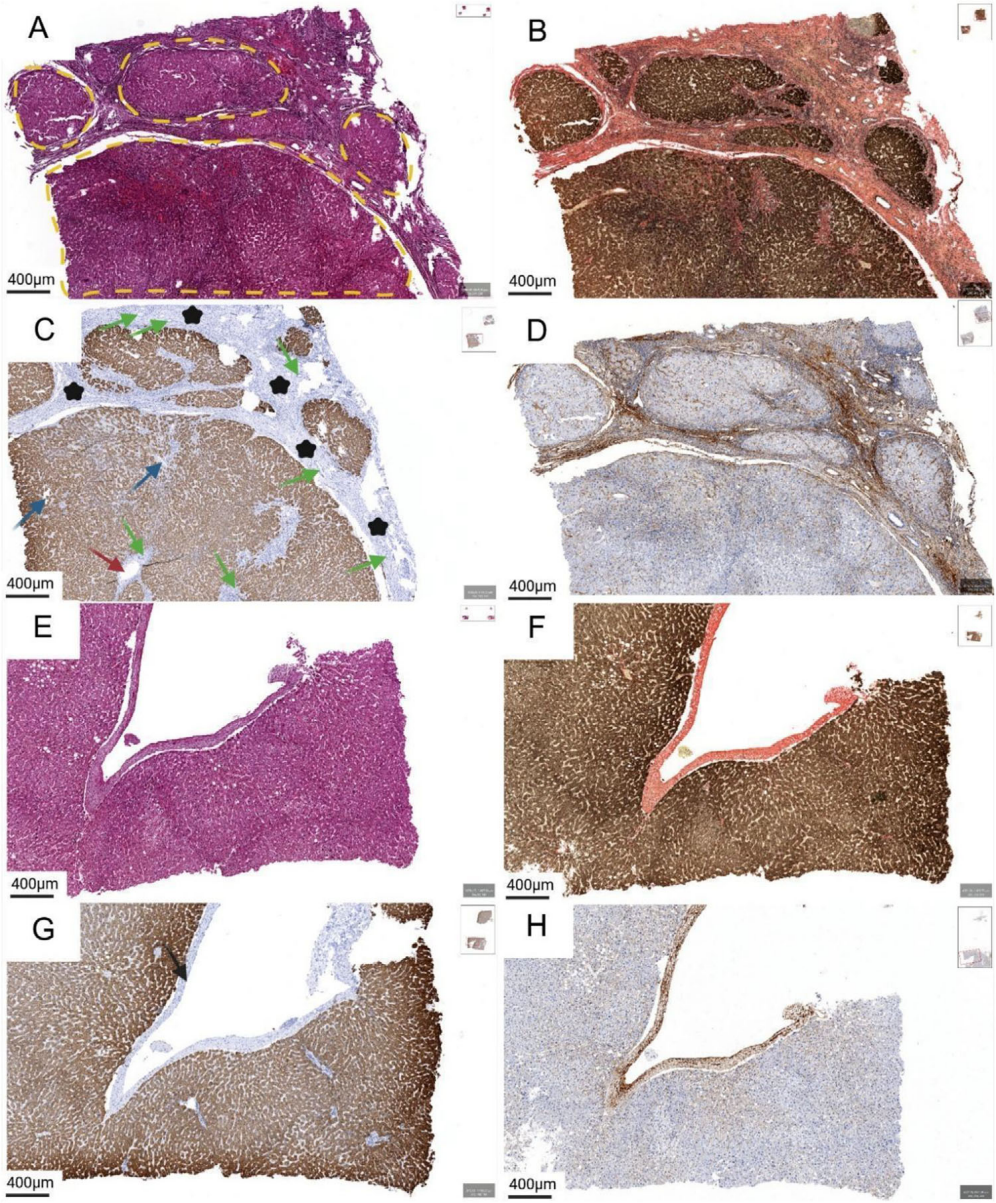
Comparative histology of cirrhotic and healthy tissue that underwent prior micro‐CT scanning. Comparative histology of cirrhotic (A–D) and normal (E–H) liver tissue post‐CT workflow. Stains include H&E (A, E), HepPar‐1/Sirius Red (B, F), HepPar‐1 (C, G), and Collagen IV (D, H). Antigenicity was preserved across all conditions. All images were made with 10x magnification. In image A, the parenchymatous nodules are marked with yellow dashed lines. In image C, the fibrous septa are marked with black stars. The bile ducts are marked with green arrows, the central veins with blue arrows, and the portal veins with red arrows. In image G, a large portal vein is marked with a black arrow. *Source*: own.

After micro‐CT imaging, the samples processed with Protocol D and E were rehydrated, embedded in paraffin, sectioned, and stained as described in Section 2.4. They showed comparable morphological quality. Standard H&E demonstrated intact cellular morphology, while Sirius Red confirmed collagen distribution. Immunohistochemistry for HepPar‐1 (hepatocyte marker) and Collagen IV (extracellular matrix marker) revealed strong, specific staining comparable to conventionally processed tissue (Table S1 and Figure 6). These results indicate that the optimized pipeline preserves antigenicity in both healthy and cirrhotic samples and is compatible with routine histopathological evaluation.

### 3.4 Compatibility With Downstream Molecular Testing

#### 3.4.1 qPCR Analysis

**FIGURE 7:**
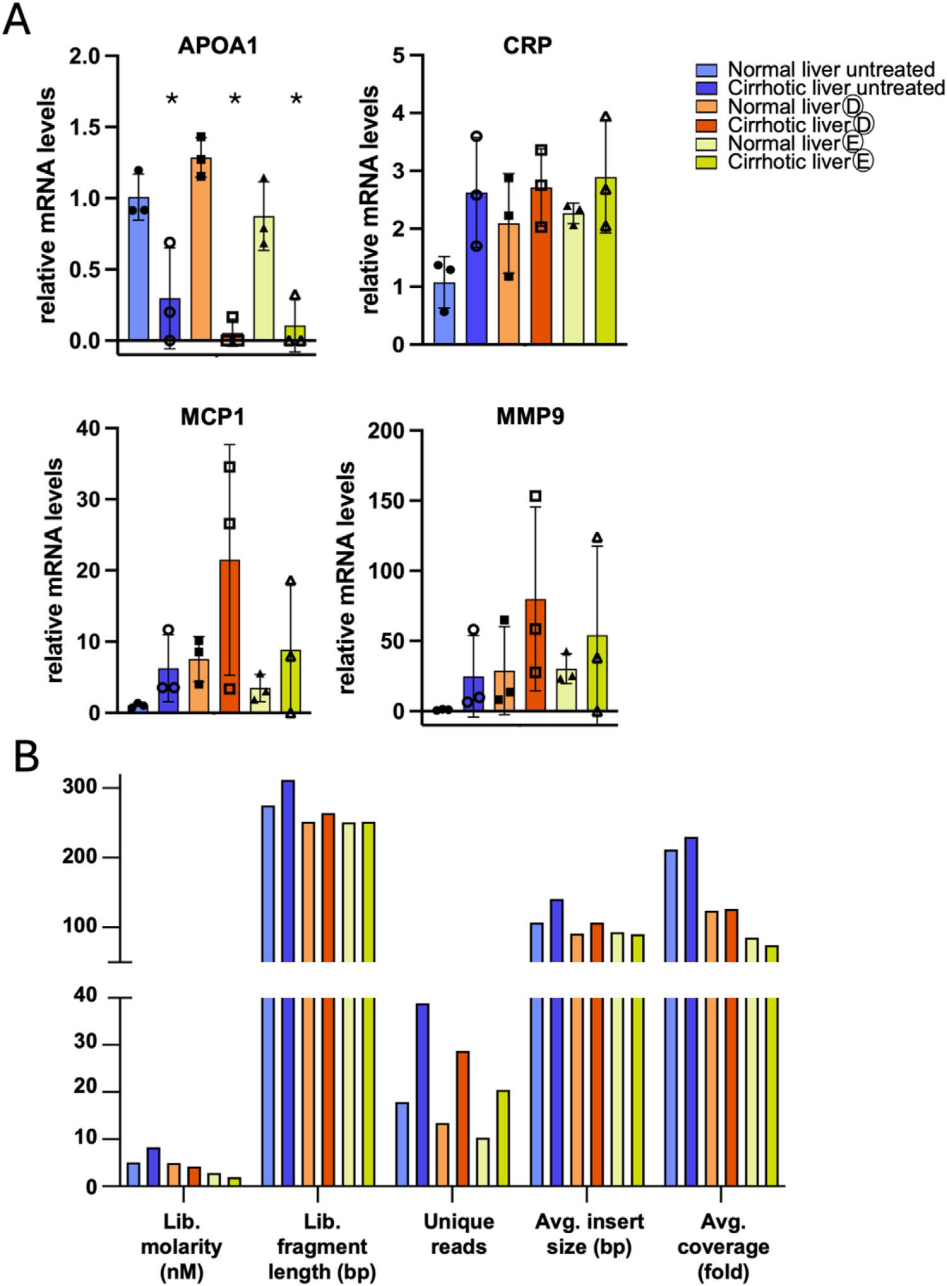
Compatibility of protocols D and E with downstream molecular analyses. (A) Relative mRNA expression of APOA1, CRP, MCP1/CCL2, and MMP9 determined by quantitative PCR in untreated normal and cirrhotic liver tissue and corresponding samples processed using protocol D or protocol E. Gene expression levels were calculated with the 2^−ΔΔCT^ method after normalization to 36b4. *n* = 3; *p <0.05 by ordinary one‐way Anova corrected for multiple comparisons using Tukey Test. (B) Comparison of targeted next‐generation sequencing (NGS) quality metrics between untreated normal and cirrhotic FFPE tissue and corresponding samples processed using protocol D and protocol E. Displayed parameters include library molarity in nM, library fragment length in bps, unique read rate as counts, average insert size in bps, and average sequencing coverage (fold). *Source*: own.

Cirrhosis is characterized by progressive fibrosis, chronic inflammation, and immune cell infiltration. Three key mediators of this process, C‐reactive protein (CRP), matrix metalloproteinase 9 (MMP9) [35, 36, 37, 38], and monocyte chemoattractant protein‐1 (MCP1/CCL2) [39, 40], are consistently upregulated in cirrhotic liver tissue. MCP1, secreted by hepatocytes, stellate cells, and endothelial cells, plays a critical role in recruiting monocytes to the liver, amplifying inflammatory and fibrotic signaling [40]. Elevated levels of CRP, MMP9, and MCP1 have been observed in experimental models and human cirrhotic samples, where their expression correlates with inflammation and disease severity [39, 40]. APOA1 encodes apolipoprotein A‐I, the principal protein component of high‐density lipoprotein (HDL). APOA1 is synthesized primarily by hepatocytes in the liver and secreted into the bloodstream, reflecting the central role of hepatocytes in the production of plasma secretory proteins. In cirrhotic liver tissue, hepatocyte function and abundance are compromised, resulting in reduced APOA1 expression [35, 36, 37, 38, 41]. Both tissue preparation protocols preserved the expected expression patterns, including decreased APOA1 and increased CRP, MCP1, and MMP9 expression in cirrhotic compared with normal liver tissue (Figure 7 and Table S2).

#### 3.4.2 NGS Analysis

Next‐generation sequencing (NGS) metrics showed that both lyophilization‐based protocols (D and E) affected library generation efficiency relative to the formalin‐fixed reference samples, albeit to different extents (Figure 7B and Table 1). Protocol D generally retained sequencing performance more effectively, whereas Protocol E showed a more pronounced reduction in library quality metrics, particularly in library molarity, mean insert size, unique read rate, and sequencing coverage.

Despite these reductions, fragment length remained largely preserved in both protocols. Library molarity and fragment length were well within the expected quality range (min. required library molarity: 1 nM (for our samples: >5 pM); fragment length: 205–450 bp). The average insert size remained almost unchanged, whereas the unique reads and the coverage were slightly reduced in specimens that have undergone protocol D and even more after protocol E (expected coverage: 200‐500‐fold (for our samples: >200‐fold)). Nonetheless, the workflow met all QC criteria for standard NGS panel sequencing [42], demonstrating that both workflows remain suitable for targeted NGS applications. Nevertheless, the superior library performance achieved with Protocol D suggests that it is the preferred workflow when preserving sequencing quality is a priority.

## 4 Discussion

This study establishes a non‐destructive, multimodal workflow that integrates high‐resolution micro‐CT imaging upstream of routine histopathology and molecular analysis within the same human liver specimens. The principal contribution of this study is not the introduction of a new imaging modality or a novel tissue preparation method in isolation, but the establishment of an integrated and validated multimodal workflow. By combining snap‐freezing, lyophilization, laboratory micro‐CT imaging, histological processing, immunohistochemistry, RNA‐based analyses, and targeted next‐generation sequencing in a single sequential protocol, the proposed workflow enables a comprehensive structural and molecular characterization of the same tissue specimen. With an optimized freeze‐dry‐based preparation, we demonstrate that 3D imaging of parenchymal, vascular, and fibrotic structures proceeds without compromising subsequent histology, immunohistochemistry, RNA integrity for qPCR, or DNA suitability for targeted NGS. Rather than replacing conventional histology, micro‐CT functions as a complementary modality that provides volumetric architectural context prior to irreversible sectioning [4, 7, 25].

A central advantage of this approach is its ability to capture spatial relationships that are only partially accessible in two‐dimensional histological sections [42]. Liver fibrosis is a heterogeneous and spatially complex process, and section‐based assessment is prone to sampling bias [9, 42]. Micro‐CT enables arbitrary virtual re‐slicing and visualization of fibrotic septa, including continuity, branching patterns, and their relationships to vascular structures within intact tissue volumes. In this proof‐of‐concept analysis, 3D segmentation reveals a marked difference in the fibrotic tissue fraction between non‐cirrhotic and cirrhotic liver samples, consistent with conventional histological assessment [26, 42], and additionally provides spatial information on the irregular, non‐linear interfaces between fibrous septa and parenchyma.

Beyond fibrosis quantification, micro‐CT offers particular value for assessing vascular remodeling. 3D visualization of portal and central veins, sinusoidal networks, and their disruption or continuity enables structural interrogation of vascular injury, congestion, and parenchymal extinction processes that are difficult to infer reliably from two‐dimensional sections alone [43, 44]. In this context, micro‐CT provides a structural framework for investigating pathogenetic concepts of vascular remodeling in chronic liver disease, including the spatial relationships between fibrotic expansion and vascular compromise [43, 45].

Compared with established two‐dimensional quantitative fibrosis approaches (Table 2), the presented workflow addresses a fundamentally different question. While 2D techniques are well‐suited for high‐throughput quantification across large cohorts [26], they remain limited to sectional representations. In contrast, micro‐CT enables direct 3D assessment of tissue connectivity, continuity, and spatial architecture [2, 3, 6]. This distinction is particularly relevant for research questions that focus on architectural remodeling, vascular topology, or spatial heterogeneity, rather than on population‐level fibrosis quantification alone [43, 45, 46].

**TABLE 2:** Overview of representative micro‐CT preparation and imaging workflows, highlighting contrast performance, compatibility with downstream analyses, laboratory accessibility, and their principal application.

Approach	Representative methods	Primary application	Soft‐tissue contrast	Histology after imaging	Molecular compatibility	Availability	Representative references
Diffusible iodine‐based staining (diceCT)	Lugol's iodine (I_2_KI), I_2_E	Contrast‐enhanced laboratory micro‐CT	High	Generally feasible after destaining; tissue shrinkage may occur	Variable depending on protocol	High	[11, 15, 20]
Heavy‐metal staining	Phosphotungstic acid (PTA), phosphomolybdic acid (PMA)	High‐resolution soft‐tissue imaging	High–Very high	Compatible	Limited evidence	High	[5, 15]
Osmium tetroxide staining	Osmium tetroxide	Ultrastructural and high‐contrast imaging	Excellent	Limited routine applicability	Generally unsuitable for routine molecular workflows	Moderate	[5]
Propagation‐based synchrotron phase‐contrast micro‐CT	Virtual histology without exogenous contrast	3D virtual histology	Excellent	Excellent	Excellent	Low (specialized synchrotron facilities)	[12, 13, 17, 18, 47]
Correlative microCT–histology workflows	Radiopaque fiducial markers, automated image registration	Multimodal image correlation	High	Excellent	Not the primary focus	Moderate	[14]
Lyophilization‐assisted multimodal workflow (present study)	Freeze‐dry protocol with micro‐CT	Sequential imaging, histology, and molecular profiling	High	Demonstrated	Demonstrated (IHC, RNA, qPCR, and targeted NGS)	Moderate	Present study

An additional strength of the proposed workflow is the preservation of tissue for downstream multimodal analyses. Immunohistochemical staining demonstrates preserved antigenicity, consistent with prior observations on the robustness of standard epitopes following optimized tissue processing [24]. RNA extracted from scanned tissue remains suitable for quantitative PCR, with expected upregulation of inflammatory mediators in cirrhotic tissue [39, 40, 48]. Furthermore, both lyophilization‐based workflows enable successful targeted NGS. Although Protocol E exhibits reduced library generation efficiency and sequencing performance compared with Protocol D, both protocols yield sequencing libraries that can be successfully processed for downstream targeted NGS. These findings align with established evidence that pre‐analytical processing can influence sequencing metrics while preserving analytical validity when quality thresholds are met [49].

From a practical perspective, the current workflow is not intended for routine diagnostic pathology. The time and resource requirements of high‐resolution micro‐CT imaging confine its primary utility to hypothesis‐driven research applications. However, this limitation is offset by its particular suitability for tissue‐scarce scenarios, biobanking contexts, and translational studies where maximizing information yield from limited material is critical [23, 25, 50]. The deliberate use of small tissue volumes reflects this focus, prioritizing spatial interrogation and multimodal reuse over screening for focal lesions.

An important consideration of the proposed workflow is the processing‐related tissue shrinkage associated with dehydration and, in particular, lyophilization. Our additional experiments demonstrated that while these steps result in a measurable reduction in specimen volume, the overall tissue architecture and the spatial relationships between parenchyma, fibrotic septa, and vascular structures remain largely preserved. Consequently, the workflow is well‐suited for qualitative assessment and relative volumetric analyses, although absolute morphometric measurements should account for this systematic shrinkage. Interestingly, the cirrhotic specimen exhibited less shape deformation than the non‐cirrhotic tissue, which, to our understanding, reflects the increased mechanical stability conferred by the collagen‐rich fibrotic extracellular matrix. Although this observation requires validation in larger cohorts, it suggests that tissue composition may influence the extent of processing‐related deformation.

In addition, lower‐magnification micro‐CT imaging proved sufficient for most applications related to architectural assessment and target selection for downstream analyses, while substantially reducing acquisition time compared with higher magnifications. This observation is consistent with prior work demonstrating that mesoscopic resolution is sufficient to capture diagnostically and biologically relevant tissue architecture [3, 6].

Future directions should include systematic reconstruction of vascular trees across diverse pathological entities (e.g., focal nodular hyperplasia, hepatocellular carcinoma, hepatocellular adenoma, and cirrhosis). Establishing entity‐specific 3D vascular atlases could not only refine morphological classification but also uncover vascular signatures predictive of biological behavior. In parallel, integrating micro‐CT datasets with spatial transcriptomics, proteomics, and metabolomics will enable true morphomolecular correlation. Image–omics fusion, for instance, linking vascular remodeling patterns to local gene expression, has the potential to identify pathways that drive fibrogenesis, tumor vascularization, or regenerative nodules. Such multimodal approaches could advance biomarker discovery, enable digital twins of liver disease progression, and ultimately support precision diagnostics and therapy stratification.

## 5 Conclusion

Beyond optimizing image quality, the present study establishes a practical multimodal tissue‐preparation workflow that enables high‐resolution laboratory micro‐CT imaging, along with comprehensive downstream histopathological and molecular analyses, from the same tissue specimen. In summary, optimized micro‐CT imaging can be integrated into pathology research workflows as a non‐destructive, information‐enhancing step that bridges 3D morphology with conventional histology and molecular profiling. By enabling volumetric architectural insight while preserving downstream analytical compatibility, this approach offers a powerful adjunct for morphomolecular investigations of liver disease and other pathologies characterized by complex spatial remodeling.

## Author Contributions

**Conceptualization**: KS, CL, UM, and BV; **Data curation**: KS, LČĆ, JV, SS, and MW; **Formal analysis**: KS; **Funding acquisition**: CL and UM; **Investigation**: KS, LČĆ, JV, SS, MW, and RS; **Methodology**: KS; **Project administration, Resources, and Software**: UM, KP, and BV; **Supervision and Validation**: CL, KP, UM, and BV; **Visualization**: KS, LČĆ, and JV; **Writing – original draft**: KS, LČĆ, JV, MW, RS, UM, KP, and BV; **Writing – review & editing**: KS, LČĆ, JV, SS, MW, RS, CL, KK, MP, GRB, IRW, UM, KP, and BV.

## Generative AI Statement

Generative AI was used solely for language refinement and editing purposes. All content, ideas, and interpretations are original and were developed independently by the authors.

## Conflicts of Interest

The authors declare no conflict of interest.

## Supporting information




**Supporting File 1**: advs77406‐sup‐0001‐SuppMat.docx.

**Supporting File 2**: advs77406‐sup‐0002‐VideoS1.mp4.

## Data Availability

The data supporting the findings of this study are presented in the article. Source files are also available from the corresponding authors upon reasonable request.
