# Supplementary material for: An Optimized Freeze‐Dry Multimodal Workflow for Sequential Micro‐CT Imaging, Histology, and Molecular Profiling: A Use Case in Human Liver Fibrosis
Source: Adv Sci (Weinh). 2026 Aug 28:e77406. Online ahead of print. doi: 10.1002/advs.77406 (PMC13525348; doi:10.1002/advs.77406)
Supplement: Supplementary file 1 [file ADVS-9999-e77406-s002.docx]

***Original research***

**An Optimized Freeze-Dry Multimodal Workflow for Sequential Micro-CT Imaging, Histology, and Molecular Profiling: A Use Case in Human Liver Fibrosis**

Kristijan Skok^1,2^, Laura Činč Ćurić^2^, Jernej Vajda^2^, Silvia Schauer^1^, Martin Wagner^3,7,8^, Rudolf Stauber^3^, Carolin Lackner^1,4^, Karl Kashofer^1^, Markus Plass^1^, Giovanny Rodriguez Blanco^5^, Ian R. Wanless^6^, Uroš Maver^2^, Katrin Panzitt^1,7,8*^ and Boštjan Vihar^2*^

| ^1^ | Diagnostic and Research Institute of Pathology, Medical University of Graz, 8010 Graz, Austria |
| --- | --- |
| ^2^ | Institute of Biomedical Sciences, Faculty of Medicine, University of Maribor, 2000 Maribor, Slovenia |
| ^3^ | Division of Gastroenterology and Hepatology, Department of Internal Medicine, Medical University of Graz, 8036 Graz, Austria |
| ^4^ | ZLP - Zentrum für Leber- und Pankreaspathologie, ADK Diagnostics, Panikengasse 45, 1160 Vienna, Austria |
| ^5^ | Clinical Institute of Medical and Chemical Laboratory Diagnostics, Medical University of Graz, Auenbruggerplatz 15, 8036 Graz, Austria. |
| ^6^ | Department of Pathology, Queen Elizabeth II Health Sciences Centre, Dalhousie University, 1276 South Park Street, Halifax, NS, Canada. |
| ^7^ | Research Unit for Translational Nuclear Receptor Research, Stiftingtalstraße 24, 8010 Graz, Austria. |
| ^8^ | BioTechMed Graz, Austria. |
|  |  |

^*^**Corresponding authors**

Assist. Professor Boštjan Vihar, PhD,

Institute of Biomedical Sciences

Faculty of Medicine, University of Maribor,

Taborska ulica 8, SI-2000 Maribor, Slovenia

[bostjan.vihar@um.si](mailto:bostjan.vihar@um.si)

Phone: +386 2 2345 878

Katrin Panzitt, PhD

Diagnostic and Research Institute of Pathology,

Medical University of Graz,

Neue Stiftingtalstraße 6, 8010 Graz, Austria

[katrin.panzitt@medunigraz.at](mailto:katrin.panzitt@medunigraz.at)

Phone: +43 316 385 71779

**Figure S1: Laboratory micro-CT instrumentation and sample mounting.**

(A) Close-up of the sample mounted on the rotation stage inside the imaging chamber prior to scanning. (B) Sample and holder mounted in the 3D x-ray microscope (Zeiss Xradaia 620 Versa) for micro CT acquisition.

**Figure S2: DNA integrity number (DIN).**

(A) DIN values. (B) DIN comparison between the healthy and cirrhotic liver samples.

**Figure S3: RNA integrity number (RIN).**

**Table S1: List of utilized stains**

| **Stain name** | **Source** | **Clone** | **Dillution** |
| --- | --- | --- | --- |
| HepPar-1 (IHC) | DAKO | M7158 | 1:200 |
| Collagen IV (IHC) | Biozol Diagnostica | Clone Isotype IgG1 | RTU |

Legend: IHC – immunohistochemistry.

**Table S2: List of used primers for PCR**

| Name | Fwd | Rev |
| --- | --- | --- |
| IL1β | 5´CCA CAG ACC TTC CAG GAG AA 3´ | 5´CGT GCA CAT AAG CCT CGT TA 3´ |
| MCP1 | 5´AGA ATC ACC AGC AGC AAG TGT CC3´ | 5´TCC TGA ACC CAC TTC TGC TTG G 3´ |
| APOA1 | 5’ACTGTGTACGTGGATGTGCTCAA 3’ | 5’AGTTGTCAAGGAGCTTTAGGTTTAGC 3’ |
| CRP | 5’ AACGAAGCCTCTCAAAGCCTT 3’ | 5’ CTCTTGGTGGCATACGAGAAAAT 3’ |
| MMP9 | 5’TGTACCGCTATGGTTACACTCG3’ | 5’GGCAGGGACAGTTGCTTCT3’ |
| 36b4 | 5’GTCACTGTGCCAGCTCAGAA3’ | 5’TCAATGGTGCCTCTGGAGAT3’ |

Legend: IL1β, interleukin-1 beta; MCP1 (CCL2), monocyte chemoattractant protein-1; APOA1, apolipoprotein A1; CRP, C-reactive protein; MMP9, matrix metalloproteinase-9; 36B4 (RPLP0), housekeeping gene used for normalization.

**Video S1. Micro-CT scanning and three-dimensional segmentation of human liver tissue.**

Skok, K., Laura, Č. Ć., Vajda, J., Silvia, S., Martin, W., Stauber, R., Lackner, C., Kashofer, K., Plass, M., Rodriguez Blanco, G., Ian, R. W., Maver, U., Katrin, P.& Vihar, B. (2026, August 10). Video S1. Micro-CT scanning and three-dimensional segmentation of human liver tissue. (Version 1) [Video recording]. Zenodo. <https://doi.org/10.5281/zenodo.21872142>
